# ECDC’s latest publications

**DOI:** 10.2807/1560-7917.ES.2017.22.13.30497

**Published:** 2017-03-30

**Authors:** Eurosurveillance editorial team

**Affiliations:** 1 European Centre for Disease Prevention and Control (ECDC), Stockholm, Sweden

**Keywords:** infectious diseases, public health, Europe

**Rapid risk assessments**

****

Cluster of new Salmonella serotype cases in four EU Member States

Date of publication: 20 March 2017

http://ecdc.europa.eu/en/publications/_layouts/forms/Publication_DispForm.aspx?List=4f55ad51-4aed-4d32-b960-af70113dbb90&ID=1664

 

Yellow fever among travellers returning from South America

Date of publication: 15 March 2017

http://ecdc.europa.eu/en/publications/_layouts/forms/Publication_DispForm.aspx?List=4f55ad51-4aed-4d32-b960-af70113dbb90&ID=1657

 

Genetic evolution of influenza A(H7N9) virus in China - implications for public health. Sixth update, 9 March 2017

Date of publication: 10 March 2017

http://ecdc.europa.eu/en/publications/_layouts/forms/Publication_DispForm.aspx?List=4f55ad51-4aed-4d32-b960-af70113dbb90&ID=1655

 

Ongoing outbreak of measles in Romania, risk of spread and epidemiological situation in EU/EEA countries

Date of publication: 7 March 2017

http://ecdc.europa.eu/en/publications/_layouts/forms/Publication_DispForm.aspx?List=4f55ad51-4aed-4d32-b960-af70113dbb90&ID=1653

 

Joint rapid outbreak assessment: multi-country outbreak of *Salmonella* Enteritidis phage type 8, MLVA profile 2-9-7-3-2 and 2-9-6-3-2 infections

Date of publication: 7 March 2017

http://ecdc.europa.eu/en/publications/_layouts/forms/Publication_DispForm.aspx?List=4f55ad51-4aed-4d32-b960-af70113dbb90&ID=1654

 

Hepatitis A outbreaks in the EU/EEA mostly affecting men who have sex with men

Date of publication: 23 February 2017

http://ecdc.europa.eu/en/publications/_layouts/forms/Publication_DispForm.aspx?List=4f55ad51-4aed-4d32-b960-af70113dbb90&ID=1648

 

Re-emerging multi-country WGS-defined outbreak of *Salmonella* Enteritidis, MLVA type 2-12-7-3-2 and 2-14-7-3-2

Date of publication: 6 February 2017

http://ecdc.europa.eu/en/publications/_layouts/forms/Publication_DispForm.aspx?List=4f55ad51-4aed-4d32-b960-af70113dbb90&ID=1640

 

Human infection with avian influenza A(H7N9) virus

Date of publication: 27 January 2017

http://ecdc.europa.eu/en/publications/_layouts/forms/Publication_DispForm.aspx?List=4f55ad51-4aed-4d32-b960-af70113dbb90&ID=1634

 

Increase in *Salmonella* Stourbridge infections in Germany during 2016

Date of publication: 27 January 2017

http://ecdc.europa.eu/en/publications/_layouts/forms/Publication_DispForm.aspx?List=4f55ad51-4aed-4d32-b960-af70113dbb90&ID=1633

 

Outbreak of yellow fever in Brazil, 25 January 2017

Date of publication: 26 January 2017

http://ecdc.europa.eu/en/publications/_layouts/forms/Publication_DispForm.aspx?List=4f55ad51-4aed-4d32-b960-af70113dbb90&ID=1630

 

**Technical reports**

 

Effectiveness and cost-effectiveness of antenatal screening for HIV, hepatitis B, syphilis and rubella susceptibility

Date of publication: 21 March 2017

http://ecdc.europa.eu/en/publications/_layouts/forms/Publication_DispForm.aspx?List=4f55ad51-4aed-4d32-b960-af70113dbb90&ID=1665

 

Antenatal screening approaches effective in preventing MTCT of HIV, HBV, syphilis and rubella in vulnerable populations

Date of publication: 21 March 2017

http://ecdc.europa.eu/en/publications/_layouts/forms/Publication_DispForm.aspx?List=4f55ad51-4aed-4d32-b960-af70113dbb90&ID=1666

 

A literature review on community and institutional preparedness synergies

Date of publication: 23 February 2017

http://ecdc.europa.eu/en/publications/_layouts/forms/Publication_DispForm.aspx?List=4f55ad51-4aed-4d32-b960-af70113dbb90&ID=1645

 

Proposals for EU guidelines on the prudent use of antimicrobials in humans

Date of publication: 20 February 2017

http://ecdc.europa.eu/en/publications/_layouts/forms/Publication_DispForm.aspx?List=4f55ad51-4aed-4d32-b960-af70113dbb90&ID=1643

 

European Gonococcal Antimicrobial Surveillance Programme external quality assessment scheme for *Neisseria gonorrhoeae* antimicrobial susceptibility testing

Date of publication: 20 February 2017

http://ecdc.europa.eu/en/publications/_layouts/forms/Publication_DispForm.aspx?List=4f55ad51-4aed-4d32-b960-af70113dbb90&ID=1638

 

**Scientific Advice**

  

Antenatal screening for HIV, hepatitis B, syphilis and rubella susceptibility in the EU/EEA – addressing the vulnerable populations

Date of publication: 21 March 2017

http://ecdc.europa.eu/en/publications/_layouts/forms/Publication_DispForm.aspx?List=4f55ad51-4aed-4d32-b960-af70113dbb90&ID=1667

 

**Special report**

 

The status of the HIV response in the European Union/European Economic Area, 2016

Date of publication: 30 January 2017

http://ecdc.europa.eu/en/publications/_layouts/forms/Publication_DispForm.aspx?List=4f55ad51-4aed-4d32-b960-af70113dbb90&ID=1635

 

**Technical document**

 

ECDC study protocol for genomic-based surveillance of carbapenem-resistant and/or colistin-resistant Enterobacteriaceae at the EU level

Date of publication: 16 March 2017

http://ecdc.europa.eu/en/publications/_layouts/forms/Publication_DispForm.aspx?List=4f55ad51-4aed-4d32-b960-af70113dbb90&ID=1658

 

Catalogue of infection control and hospital hygiene courses in the European Union

Date of publication: 30 January 2017

http://ecdc.europa.eu/en/publications/_layouts/forms/Publication_DispForm.aspx?List=4f55ad51-4aed-4d32-b960-af70113dbb90&ID=1636

 

**Mission report**

 

Exploring opportunities for support in healthcare-associated infections (HAI) - Romania, 4-7 July 2016

Date of publication: 19 January 2017

http://ecdc.europa.eu/en/publications/_layouts/forms/Publication_DispForm.aspx?List=4f55ad51-4aed-4d32-b960-af70113dbb90&ID=1628

 

**Surveillance reports**

 

Tuberculosis surveillance and monitoring in Europe, 2017

Date of publication: 20 March 2017

http://ecdc.europa.eu/en/publications/_layouts/forms/Publication_DispForm.aspx?List=4f55ad51-4aed-4d32-b960-af70113dbb90&ID=1663

 

Molecular typing for surveillance of multidrug-resistant tuberculosis in the EU/EEA

Date of publication: 6 March 2017

http://ecdc.europa.eu/en/publications/_layouts/forms/Publication_DispForm.aspx?List=4f55ad51-4aed-4d32-b960-af70113dbb90&ID=1651

 

The European Union summary report on antimicrobial resistance in zoonotic and indicator bacteria from humans, animals and food in 2015

Date of publication: 22 February 2017

http://ecdc.europa.eu/en/publications/_layouts/forms/Publication_DispForm.aspx?List=4f55ad51-4aed-4d32-b960-af70113dbb90&ID=1644

 

Antimicrobial resistance surveillance in Europe 2015

Date of publication: 30 January 2017

http://ecdc.europa.eu/en/publications/_layouts/forms/Publication_DispForm.aspx?List=4f55ad51-4aed-4d32-b960-af70113dbb90&ID=1637

